# Highly Efficient Autologous HIV-1 Isolation by Coculturing Macrophage With Enriched CD4^+^ T Cells From HIV-1 Patients

**DOI:** 10.3389/fviro.2022.869431

**Published:** 2022-04-07

**Authors:** Cristina Xufré, Tanía González, Lorna Leal, Charles M. Trubey, Jeffrey D. Lifson, José María Gatell, José Alcamí, Núria Climent, Felipe García, Sonsoles Sánchez-Palomino

**Affiliations:** 1 AIDS Research Group, Institut d’Investigacions Biomèdiques August Pi I Sunyer (IDIBAPS), Hospital Clinic, University of Barcelona, Barcelona, Spain; 2 Centro de Investigación Biomédica en Red (CIBER) of Infectious Diseases, Centro de Investigación Biomédica en Red de Enfermedades Infecciosas, (CIBERINFEC), Instituto de Salud Carlos III, Madrid, Spain; 3 Infectious Diseases Service, Hospital Clinic, Institut d’Investigacions Biomèdiques August Pi I Sunyer (IDIBAPS), University of Barcelona, Barcelona, Spain; 4 AIDS and Cancer Virus Program Inc., Frederick National Laboratory, Frederick, MD, United States; 5 AIDS Immunopathology Unit, National Center of Microbiology, Instituto de Salud Carlos III, Madrid, Spain

**Keywords:** autologous HIV immunogen, HIV isolation, HIV therapeutic vaccine, CD4^+^ T cells, coculture, monocyte-derived macrophages (MDM)

## Abstract

We described a novel HIV autologous isolation method based in coculturing macrophages and CD4^+^T-cell-enriched fractions from peripheral blood collected from antiretroviral-treated (ART) HIV patients. This method allows the isolation of high viral titers of autologous viruses, over 10^10^HIV RNA copies/ml, and reduces the time required to produce necessary amounts for virus for use as antigens presented by monocyte-derived myeloid cells in HIV therapeutic vaccine approaches. By applying these high titer and autologous virus produced in the patient-derived cells, we intended to elicit a boost of the immunological system response in HIV therapeutic vaccines in clinical trials.

## INTRODUCTION

HIV infection currently constitutes one of the main worldwide pandemics with around 1.5 million new cases in 2020 and 37.7 million people living with HIV but only 27.5 million people have access to antiretroviral therapy (ART) (1). HIV targets CD4^+^ cells involved in the immune response, mainly CD4^+^ T cells and monocyte/macrophages (2, 3).

Different combination ART regimens have been implemented since 1996, representing a major and significant clinical advancement in reducing HIV morbidity and mortality, as well as re-establishing the T-cell protective immunity against opportunistic pathogens (4). These therapies rendered a plasma virus load (pVL) of less than 50 HIV RNA copies/ml and increased CD4 cell count number to reach roughly normal values in many treated individuals. Therefore, ART has transformed HIV infection from being a fatal disease to a chronic infection with an excellent quality of life (5). However, ART has a major limitation because HIV-infected patients must be treated for life to prevent HIV rebound from the viral reservoir, virus which persists despite extended virus suppression on ART and can give rise to a recrudescent progressive infection if ART is stopped (6, 7).

In this context, a therapeutic anti-HIV vaccine, capable of targeting cells harboring the viral reservoir and maintaining control of any residual virus after cessation of ART, remains a major challenge. One of the most promising approaches to achieve a therapeutic vaccine is the development of *ex vivo* antigen pulsed monocyte-derived dendritic cells (MDDCs) for use as *in vivo* antigen-presenting cells to promote cytotoxic and helper T-cell anti-HIV responses (8). Dendritic cells are professional antigen-presenting cells (9) connecting the innate and adaptive immune responses (10). Therapeutic vaccines based on *ex vivo* antigen pulsing of autologous MDDC have been previously used against cancer (11). Nevertheless, only modest improvements have been reported so far for HIV treatment (12), with arguably the most promising result achieved to date for a MDDC-based HIV therapeutic vaccine developed in our group enabling >95% VL reduction for 1 year in recipient patients (13). DC-based vaccine efficacy is hard to foresee due to variations in the immunogens chosen for loading DCs with autologous virus immunogens, how this immunogen is inactivated, whether ART-naïve or ART-experienced patients are enrolled for the clinical trials conducted, the criteria used to evaluate vaccine elicited responses, or the techniques for DC culture, viral isolation, and expansion (Supplementary Table S1) (12–18).

ART-treated HIV patients were enrolled in one of the clinical trials led by our group to test a novel dendritic cell-based anti-HIV vaccine (protocol code DCV3/RISVac04) (19). ART-treated HIV patients usually carry replication-competent HIV in only a scarce proportion from their peripheral blood cells, which can represent a challenge in the generation of autologous viral stocks from CD4^+^ T cells (20). To facilitate autologous viral isolation and prepare stocks for therapeutic vaccines, ART-treated HIV patients enrolled in DCV3 trial underwent a structured treatment interruption (STI). To generate autologous viral stocks, we have developed a new method based on the coculture of monocyte-derived macrophages (MDM) from peripheral blood and a fraction of CD4^+^-enriched lymphocytes. With this approach, we rescue the different autologous viral quasispecies present in both cell types that are targets of HIV infection. This method allows faster isolation of autologous HIV at high titers, thus facilitating the production of viral stocks for pulsing of dendritic cells in therapeutic vaccine protocols (21).

## MATERIAL AND METHODS

### Patients

Our DC-based clinical trial (protocol code DCV3/RISVac04) pursued a therapeutic vaccine development (EudraCT No.: 2015-001795-22). The enrolment criteria included HIV-infected individuals 18 years or older, with ≥450 CD4^+^ T cells/mm^3^, and receiving ART for at least 1 year with successful viral suppression (pVL ≤50 HIV-1 RNA copies/ml) for at least 6 months. All participants were men who have sex with men (MSM) and were recruited at the Hospital Clinic i Provincial (Barcelona, Spain). All participants signed an informed consent. The trial was approved by the ethics committee of the Hospital Clinic i Provincial and was supervised by the Institutional Committee of Ethical and Clinical Investigation (19).

### Reagents and Antibodies

Every reagent involved in vaccine preparation was sterile, endotoxin free, and classified as pharmaceutical product or elaborated under good manufacturing practice (GMP) conditions (18).

Culture and viral isolation techniques were always performed under sterile conditions in Bio II/A biosafety hood according to previously validated standard operational procedures approved at the Production and Validation Center of Advanced Therapies (Creatio, University of Barcelona, Barcelona, Spain) during the preclinical evaluation of this project.

Microbiological sterility analysis was performed at Echevarne laboratories assessing for bacteria, fungi, mycobacteria, and endotoxin level. The procedures were validated according to the monograph Ph. Eur.2.6.1. In addition, the presence of mycoplasmas was determined by an enzymatic quantification assay using the MycoAlert^®^ Mycoplasma Detection Assay(Lonza, Basel, Switzerland).

Peripheral blood mononuclear cell (PBMC) fractions were cultured in *X-VIVO* 15 medium (Lonza) supplemented with 10% AB human serum (ABHuS). To that aim, AB human plasma from anonymous healthy blood bank donors, tested negative for HBsAg, anti-HCV, anti-HIV-1 plus anti-HIV-2, HCV-RNA, HIV-1-RNA, HBV-DNA, and syphilis (TPHA), was firstly obtained (Banc de Sang i Teixits, Barcelona, Spain). AB human plasma was then converted to ABHuS for 30 min treatment at 37°C with 10 U/ml human thrombin (Tissucol Duo^®^, Baxter, IL, USA), followed by centrifugation at 6,000×*g* for 30 min. Finally, ABHuS was heat inactivated at 56°C for 30 min, centrifuged again at 6,000×*g* for 30 min, and stored frozen at −20°C until use.

MACS^®^ CD3 pure and MACS^®^ CD28 pure GMP-certified antibodies, CliniMACS CD8 and CD14 microbeads, CliniMACS LS columns, and CliniMACS PBS/EDTA buffer were from Miltenyi Biotec, Bergisch Gladbach, Germany. Pharmaceutical-grade human recombinant interleukin-2 (IL-2; Chiron Corporation, Emeryville, CA, USA) was diluted at 10^4^ UI/ml in *X-VIVO* 15 culture medium, aliquoted, and frozen at −20°C until use, according to manufacturer’s expiration date.

Cell phenotype was analyzed by flow cytometry using fluorescently labelled monoclonal antibodies recognizing CD14, CD8, CD3, CD4, and CD25 from R&D Systems (R&D Systems, MN, USA); anti-CD71 was purchased from Miltenyi Biotec. Cytometry data were captured with a FACSCANTO II (Becton-Dickinson, NJ, USA) and analyzed with v6.1 FACSDiva software (Becton-Dickinson).

VL was determined by VERSANT^®^ HIV-1 RNA 1.0 Assay (kPCR; Siemens Healthcare, Erlangen, Germany) and HIV-1 p24 antigen quantification by ELISA (INNOTEST^®^ HIV Antigen mAb Fujirebio, Fujirebio, Tokyo, Japan).

Other commonly used reagents for cell cultures consisted of the following: DMEM culture media (Lonza), fetal bovine serum (FBS;; Gibco, Thermo Fisher Scientific, Waltham, MA, USA), trypsin (Invitrogen, Thermo Fisher Scientific), and *Renilla* Luciferase Assay Stem (Promega Corporation, WI, USA).

### Autologous HIV-1 Isolation

**FIGURE 1 |:**
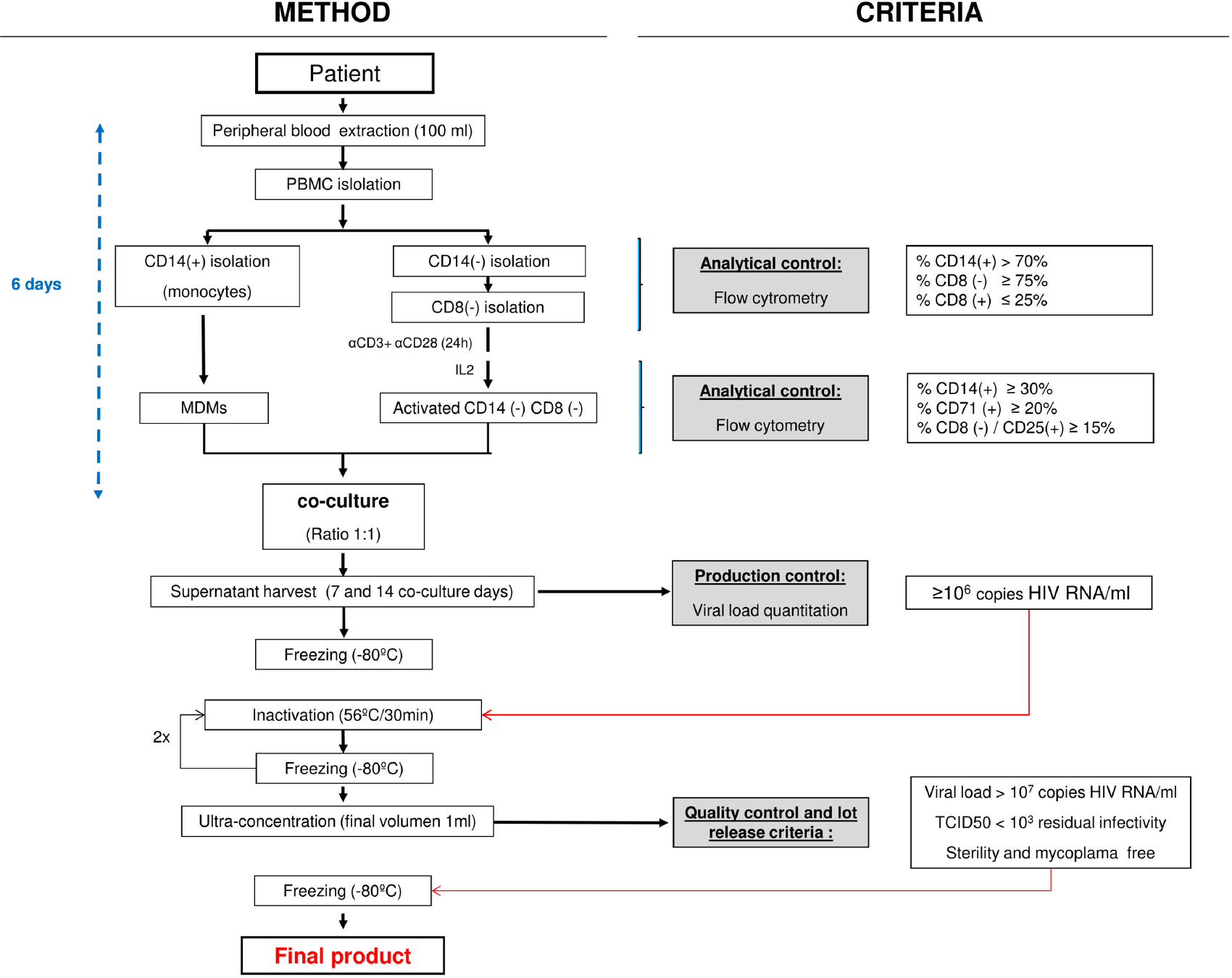
GMP HIV-1 autologous production process flowchart. Light gray boxes show the quality control steps required. Empty boxes on the right describe the Investigational Medicinal Product Dossier (IMPD)-based criteria applied. GMP, good manufacturing practice; PBMC, peripheral blood mononuclear cell; MDM, monocyte-derived macrophage.

A brief STI was performed in HIV patients participating in the trial to facilitate autologous HIV *in vitro* isolation, for pairing with autologous MDDC as the pulsed antigen-presenting cells for the therapeutic vaccine. Additionally, this approach will avoid any immunological response against allogeneic donor proteins (19). HIV-1 isolation started when viral rebound reached pVL ≥5,000 HIV-1 RNA copies/ml, as established in the Investigational Medicinal Product Dossier (IMPD) for the clinical trial (EudraCT n: 2015-001795-22, promoter’s protocol code DCV3/RIsVac04). To that aim, 100 ml of blood were obtained and PBMC purified by Ficoll centrifugation (ACCUSPIN^™^ System-Histopaque^®^-1077, Sigma Diagnostics Inc., Livonia, MI, USA) from STI patients. Viral isolation was performed by coculturing those cells arising from CD14^+^ and CD14^−^CD8^−^ fractions recovered from PBMC as described in the flowchart presented in Figure 1.

Briefly, the CD14^+^ cell fraction (monocytes) was purified from PBMC by magnetic cell sorting with CliniMACS CD14 MicroBeads (Miltenyi Biotec), according to manufacturer’s instructions. The CliniMACS device (Miltenyi Biotec) is a closed GMP system used for cell isolation. The differentiation of MDMs is carried out by placing upright (vertical laying) T75 culture flasks containing no more than 35 ml with the CD14^+^ cells seeded at 1 × 10^6^ cells/ml for 6 days in *X-VIVO* 15 culture medium supplemented with 10% ABHuS at 37°C in a fully humidified atmosphere. To minimize adherence, differentiation of MDM was performed on ultra-low attachment (ULA) flasks.

CliniMACS CD8 MicroBeads (Miltenyi Biotec) were used to select CD8^−^ cells in the CD14^−^ effluent PBMC fraction. Resulting isolated CD14^−^CD8^−^ cells were activated for 24 h using horizontal T75 ULA culture flasks coated with anti-CD3 (2 μg/ml) plus anti-CD28 (0.2 μg/ml) antibodies. These cells were seeded at 1 × 10^6^ cells/ml in *X-VIVO* 15 medium supplemented with 10% ABHuS and kept at 37°C in a fully humidified atmosphere for 5 additional days in the presence of 100 IU/ml human IL-2. After culturing, cells were washed three times with PBS before establishing CD4^+^ T-cell/MDM cell cocultures for HIV-1 isolation.

MDMs plus activated CD14^−^CD8^−^-derived cells were seeded in coculture 6 days after blood collection at 1:1 cell ratio (best cell ratio condition for HIV production) (22) at 0.5 × 10^6^ cells/ml, and supernatants were harvested at 7 and 14 days after coculture onset. These cocultures never surpassed 50 ml final volume/bottle and were upright incubated in T75 ULA flasks in *X-VIVO* 15 medium supplemented with 10% ABHuS and 100 IU/ml IL-2, as described above. Seven days after coculture onset, fresh medium replaced twothirds of the volume. Seven and 14 days collected HIV coculture supernatants were clarified by centrifugation at 2,574×*g* for 32 min and titrated before being stored frozen at ≤−70°C. When the 14-day harvest was completed, coculture flasks were discarded. Within 1 month of initial freezing, viral isolates were thawed and heat inactivated as referred below. Viral production was quantified by determining HIV-1 RNA copies/ml on supernatants by qPCR (VERSANT^®^ HIV-1 RNA 1.0 Assay kPCR, Siemens Healthcare) and HIV-1 p24 Antigen-ELISA (INNOTEST^®^ HIV Antigen mAB, Fujirebio).

Coculture supernatants exhibiting a VL titer <10^6^ HIV-1 RNA copies/ml were not used, as established in the IMPD. When both harvested coculture supernatants (day 7, day 14) were discarded, the virus isolation procedure was repeated once more with a second blood extraction for that contributing participant (Table 1).

### Heat Inactivation and Concentration of Virus

HIV-1 isolates were thawed and inactivated by two 30-min heat cycles at 56°C in an Eppendorf thermomixer (Eppendorf, Hamburg, Germany) with gentle shaking (750 rpm), interspersed by a freezing and storage period at ≤−70°C, for at least 24 h. Fully heat-inactivated isolates were concentrated by ultracentrifugation at 65,700×*g* for 28 min at 4°C in 30 ml sterile polyallomer bottles (Seton Scientific, Petaluma, CA, USA) using a T1250 Fiberlite rotor. Once ultracentrifugation finished, supernatant was discarded and viral pellets were resuspended in 1 ml sterile saline solution, poured into a1.5-ml Eppendorf polypropylene tube and ultracentrifuged a second time at 128,800×*g* for 15 min at 4°C. The supernatant was discarded and viral pellets were resuspended in 1 ml sterile saline solution. Each autologous HIV-1 virus isolate was aliquoted in 5 sterile, nonpyrogenic and screwed tubes containing 0.2 ml each and stored frozen at −70°C ≤ until used in the *ex vivo* pulsing of autologous MDMCs as immunogen for a fully autologous therapeutic vaccine.

### Residual HIV Infectivity Analysis

Residual HIV infectivity was assessed by determining tissue culture infectious dose (TCID)50/ml in a TZM-bl assay. The TZM-bl cell line (NIH AIDS Research and Reference Reagent Program, Division of AIDS, NIAID, NIH, Dr. John C. Kappes, Dr. Xiaoyun Wu and Tranzyme Inc.) is a HeLa cell line that stably expresses CD4 and CCR5, with luciferase and β-galactosidase marker genes under LTR HIV-1 promoter control. TZM-bl cells were subcultured at 37°C in a fully humidified atmosphere with 5% CO_2_ in DMEM supplemented with 10% FBS, 10 U/ml penicillin, and 10 mg/ml streptomycin. In this assay, HIV infectivity was quantified as a function of luciferase activity expressed (relative light units (RLU)/ml) before and after heat treatment. Positive viral control of infectivity was an HIV-1 BaL virus supernatant grown on MDM from *in vitro*-infected healthy donor PBMCs. Infectious titer was calculated by the Spearman and Kärber algorithm being 2.4 TCID50/ml as the lower quantification limit of this assay (23). The infectivity reduction factor (*Ri*) was determined according to the following formula: *Ri* = log_10_(*V1* × *T1*/*V2* × *T2*), being *V1* = starting volume material, *T1* = starting virus concentration, *V2* = volume after the inactivation process, and *T2* = virus concentration after inactivation (24).

### Total and Integrated HIV DNA

We quantified total and integrated HIV-1 DNA in highly enriched CD4^+^ T cells from PBMCs, using negative selection magnetic beads (STEMCELL Technologies Inc., BC, Canada). HIV DNA was amplified from CD4^+^ T-cell DNA lysates by a first round of amplification (LTR-gag amplification for total DNA and Alu-LTR amplification for integrated DNA) (24, 25), and the obtained amplicons were then reamplified with nested PCR internal specific primers for each region using TaqMan probes. HIV-1 detection sensitivity in the assay was three viral DNA copies/10^6^ CD4^+^ T cells. For each assay, the CD3 gene copy number (2 copies per cell) was determined in the same tube for accurate quantification of the total HIV DNA. The HIV/CD3 DNA ratio renders HIV DNA copies normalized for cellular diploid genome DNA equivalents. For total and integrated HIV DNA, the standard curve was elaborated with DNA lysates from serial dilutions of ACH2 cells, ranging from 3 × 10^5^ to 3 cells which carry one single HIV provirus/ACH2 cell, together with the experimental samples. HIV primers and probes were optimized to efficiently amplify and detect HIV-1 from the A, B, C, D, and A/E (CRF01) clades.

### Statistical Analysis

Correlations were performed using the Pearson correlation test with GraphPad Prism 6.

## RESULTS

### Clinical Data of Enrolled ART-Treated HIV-1 Patients Subjected to Autologous Viral Isolation

Autologous HIV-1 was isolated by coculture of MDMs differentiated from CD14^+^ PBMCs, in conjunction with enriched fractions of CD4^+^ T cells (CD14^−^CD8^−^) as detailed in the Materials and Methods section (Figure 1). Eighteen ART-treated HIV-1^+^ MSM individuals were recruited. At recruitment, when STI began, total and integrated reservoir median (IQR) were 374.2 (146.3–469.8) and 90.25 (50.44–147.3) HIV-1 DNA copies/10^6^ CD4^+^ T cells, respectively. ART interruption resulted in HIV viral load rebound in agreement with previously reported data (26). The median STI was 38 days with an IQR ranging from 29.5 to 55 days. At viral culture onset, median pVL was 56,910 (IQR, 11,850–295,250) HIV-1 RNA copies/ml, 702 (IQR, 596–804.5) CD4^+^ T cells/mm^3^, and 836 (IQR, 625.5–1053) CD8^+^ T cells/mm^3^. Additional relevant clinical data for patients enrolled in DCV3 clinical trial are provided in Table 1.

### HIV-1 Autologous Coculture of PBMC-Derived Cell Fractions

**FIGURE 2 |:**
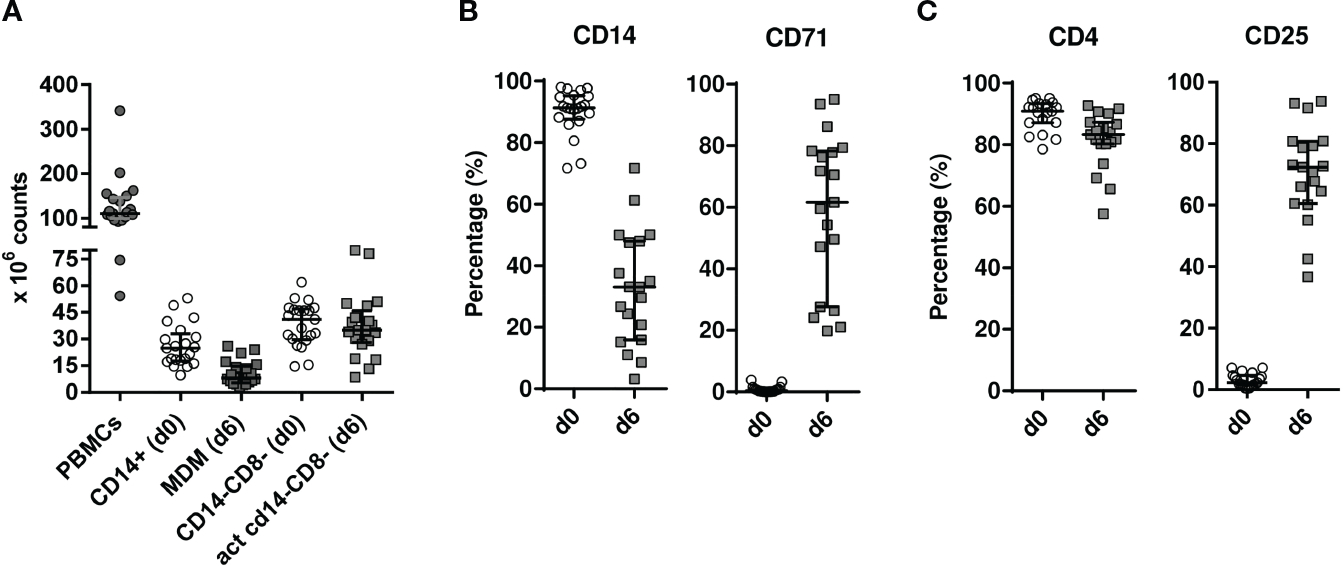
Cellularity **(A)** and flow cytometry analysis **(B, C)** of cultured cell subsets. **(A)** CD14^+^ monocytes and CD14^−^ CD8^−^ cells were magnetically isolated from PBMCs in 100 ml EDTA-treated patient blood at HIV isolation onset [day 0 (d0)]. Individual cellularity is depicted as solid circles for whole PBMC, while empty circles and solid squares are graphed for cell fractions at days 0 (d0) and 6 (d6), respectively, with median values shown as horizontal solid lines. **(B)** CD14^+^ monocyte differentiation to monocyte-derived macrophages (MDMs) was tracked by CD14 and CD71 analysis. To that goal, cells were FSC/SSC gated and analysed the monocytic window (Supplementary Figure S1A) **(C)**. On the other hand, 24-h-activated CD14^−^ CD8^−^ cells later cultured for five more days in IL-2-containing medium were plotted at d0 and d6 for CD4 and CD25 expression in FSC/SSC-gated cells restricted to a CD3^+^ lymphocytic window (Supplementary Figure S1B). PBMC, peripheral blood mononuclear cell; act, activated; FSC, forward scatter; SSC, side scatter.

Following established procedures, a mean of 110.4 × 10^6^ PBMCs (IQR, 99.56–146.5 × 10^6^ cells) were isolated, yielding high purity monocytes (CD14^+^) and CD4^+^ T-enriched lymphocytes (CD14^−^CD8^−^) cell fractions (above 90% purity). Overall, after 6 days of culture, a mean of 8.1 × 10^6^ MDM (IQR, 5.45–14.83 × 10^6^ cells) and 35 × 10^6^ activated CD4^+^-enriched T cells (IQR, 28–45.90 × 10^6^ cells) were obtained before proceeding to coculturing both populations (Figure 2A). Macrophage differentiation in culture was assessed by determining CD14 decreased and CD71 increased expressions (Figure 2B) while CD4 activation was measured by CD4^+^ and CD25^+^ T-cell expressions (Figure 2C).

### High Levels of Autologous HIV-1 Production by *In Vitro* Coculture of MDMs and Activated CD4 Lymphocytes

MDM CD14^+^ and activated CD4^+^ T cells were cocultured at a 1:1 cell ratio (22) for 14 days. Supernatants were harvested at 7 and 14 coculture days. Coculture supernatants with VLs below 10^6^ HIV-1 RNA copies/ml were discarded, and those patients with both harvests below the cutoff value just mentioned were subjected to a second 100 ml peripheral blood extraction and a new HIV isolation round (*), before ART were resumed.

**FIGURE 3 |:**
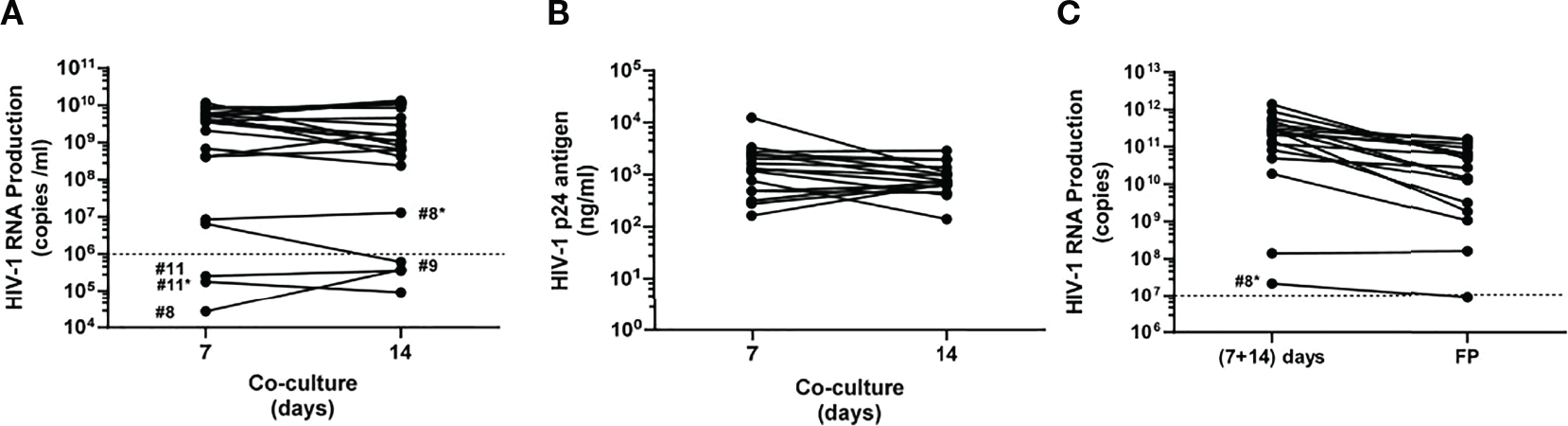
Monitoring autologous HIV-1 production. **(A)** Viral load titrated at 7 and 14 coculture days as HIV-1 RNA copies/ml in raw harvested supernatants. Dashed line shows the cutoff value according to the Investigational Medicinal Product Dossier (IMPD), meaning 10^6^ HIV-1 RNA copies/ml. **(B)** HIV-1 p24 ng antigen/ml at 7 and 14 coculture days was only determined for those supernatants exceeding 10^6^ HIV RNA copies/ml. **(C)** HIV-1 RNA copies/ml in mixed supernatants (7 + 14 coculture day harvests) versus final products (FPs). Dashed line shows 10^7^ HIV-1 RNA copies/ml as cutoff value to release immunogens, according to the Investigational Medicinal Product Dossier (IMPD). Solid circles depict the determined value for each patient at both harvesting times and are linked by a solid black line. *Second 100 ml peripheral blood extraction.

Viral titer remained quite high and constant at 7 and 14 coculture days for the majority of patients (Figure 3A; Supplementary Table S2). Nevertheless, no successful production from primary coculture supernatants was obtained for patients #8 and #11. HIV isolation was again attempted for these patients.

In summary, it was possible to generate viral stocks at high titers in 17 out of 18 enrolled patients (94.44%) after 2 weeks coculture of MDM and activated CD4^+^ T lymphocytes. These coculture supernatants were considered suitable for concentration (immunogen elaboration).

Coculture supernatants rendered median viral loads of 3.58 × 10^9^ (IQR, 2.09 × 10^8^–5.64 × 10^9^) HIV-1 RNA copies/ml and 1.05 × 10^9^ (IQR, 1.27 × 10^8^–6.60 × 10^9^) HIV-1 RNA copies/ml at 7 and 14 coculture days, respectively (Supplementary Table S2). In addition, p24 viral antigen (p24 antigen) quantitation was also determined for those primary supernatants with both harvests over 10^6^ HIV RNA copies/ml. As shown in Figure 3B and Supplementary Table S3, 1,305 (IQR, 488.5–2,575) p24 ng/ml and 771 (IQR, 632.5–1,315) HIV-1 p24 ng/ml were determined at 7 and 14 coculture days, respectively. Moreover, titers from mixing supernatants collected after 7 and 14 days in the same coculture showed a positive correlation (*r* = 0.465, *p* = 0.007) with p24 at the same harvesting periods (Supplementary Figure S2).

When possible, supernatants collected at 7 and 14 coculture days of the same donor were mixed, giving rise to 53.1 (IQR, 36.5–96.3) ml median volumes (Supplementary Table S2). Heatinactivated supernatants were concentrated by ultracentrifugation as described in the Materials and Methods section. Patient #14 mixed supernatant suffered handling problems and was subjected to a second HIV isolation procedure.

Resulting viral pellets were resuspended in 1 ml saline buffer and stored frozen until use in 5 × 200 μl, each aliquot with 3.56 × 10^10^ (IQR, 5.23 × 10^9^–8.21 × 10^10^) HIV-1 RNA copies/ml as median titer (Figure 3C). Solubilized pellets with viral titers below10^7^ HIV-1 RNA copies/ml were not released as final products to be used as immunogens.

### HIV-1 Production Correlations

**FIGURE 4 |:**
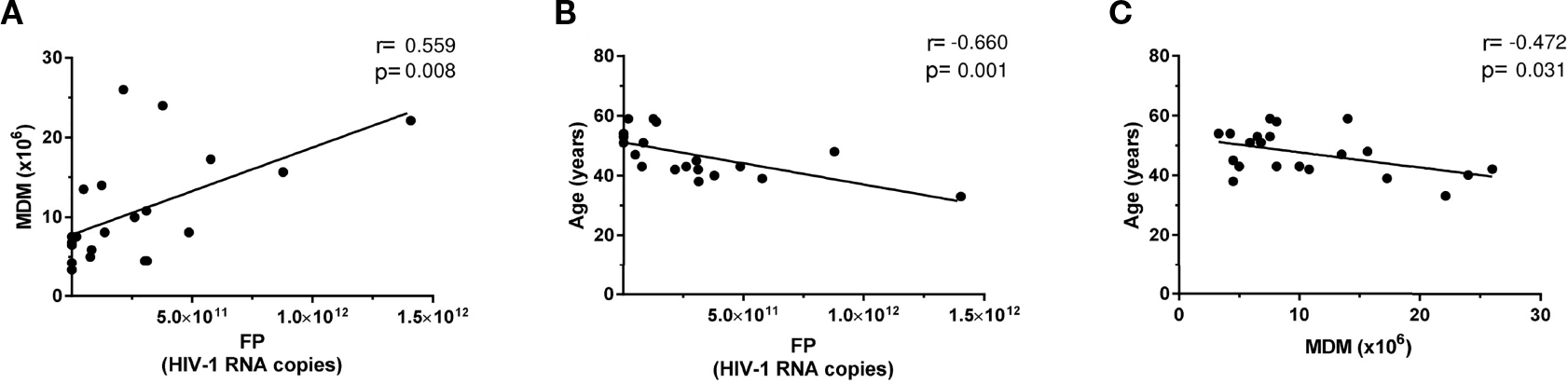
Correlation graphs on specific patient parameters. Individually depicted solid circles for each patient are graphed, showing HIV RNA copies per final product (FP) versus either **(A)** amount of monocyte-derived macrophages (MDMs) at coculture onset or **(B)** patient age. **(C)** The correlation of MDMs at coculture onset patient age. The Pearson correlation test was always applied, and resulting fitted regressions are represented by solid lines.

Data on assay correlations studied are summarized in Figure 4.

HIV-1 production positively correlated with the amount of MDM obtained (*r* = 0.559, *p* = 0.0084) (Figure 4A) and negatively correlated with patients’ age (*r* = −0.66, *p* = 0.001) (Figure 4B). Consistently, age negatively correlated with MDM (*r* = −0.472, *p* = 0.031) (Figure 4C). In addition (not plotted), a positive correlation was found between pVL at the day of 100 ml peripheral blood extraction and MDM number (*r* = 0.49, *p* = 0.025) and a trend to a positive association with respect to CD14^+^ cell number (*r* = 0.417, *p* = 0.06). However, no correlation was detected when HIV production was compared with total or integrated viral DNA. Similar correlation absence was found neither when HIV production was compared with CD14 or CD71 expression at the time of 100 ml peripheral blood extraction nor when cocultures were established.

**FIGURE 5 |:**
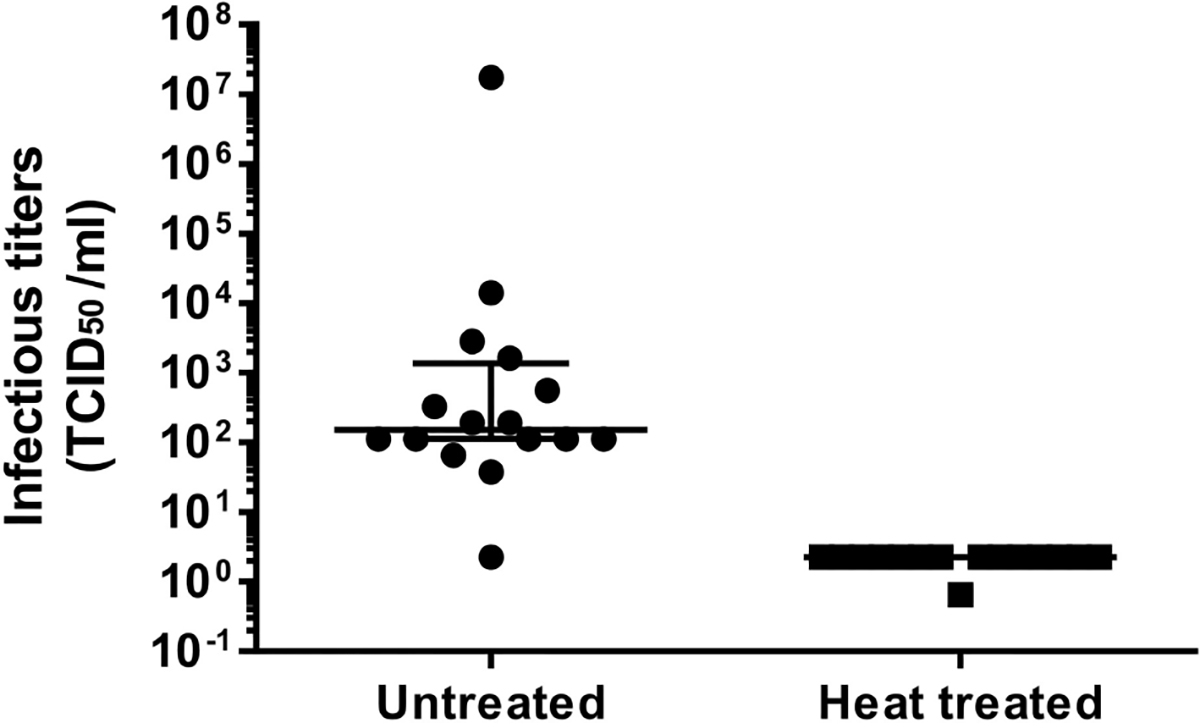
Effect of heat inactivation on HIV-1 infectivity. Supernatants were harvested at 7 and 14 days in coculture. Those over 10^6^ HIV RNA copies/ml were mixed, when possible, after inactivation. The graph shows TCID50/ml prior (left half, untreated) and after heat inactivation (right half, heat treated). Longer solid lines at both graph halves correspond to the median TCID50/ml for each group, while the upper and lower solid lines on the left indicate the 75% and 25% IQR percentiles, respectively.

Overall, we succeeded to isolate viral immunogens at very high titers required for their use in our designed autologous virus-pulsed MDDC-based therapeutic vaccine in 16 out of 18 patients (Supplementary Table S2) enrolled in this clinical trial. Patient #8 was excluded because the titer cutoff for concentrated products (10^7^ HIV-1 RNA copies/ml) was not achieved, while in patient #11, primary supernatant titers below 10^6^ HIV-1 RNA copies/ml were obtained from coculture supernatants. The viral infectivity was determined before heat inactivation and concentration displaying a median of 151.5 (IQR, 112.0–1370) TCID50/ml. Once heat was inactivated and concentrated, viral stocks were not infectious (Figure 5) as determined by TCID50, being below the assay sensitivity. Finally, sterility of viral stocks was verified as described at the IMPD. These quality control data allowed viral preparations from 16 patients to be released as final products (FPs; immunogens) to pulse autologous MDDCs in therapeutic HIV vaccine clinical trials.

## DISCUSSION

HIV-infected patients require lifelong treatment to control viral replication, and, currently, there is no effective procedure to eradicate persistent HIV from infected surviving cells (27). Two–three weeks after ART withdrawal, virus rebounds in the majority of patients from latently infected cells that constitute the so-called HIV reservoir (4, 27, 28). Our results are consistent with this established pattern (Table 1). All patients in our cohort, ART treated for no less than 1 year and in virus remission longer than 6 months, experienced a viral rebound after 38 days of STI as median value. However, the total HIV DNA median value for our recruited patients was around three times above the integrated HIV DNA. This may be explained by abundant nonintegrated viral DNA either as linear or 1- or 2-LTR circles (not determined here). In four participants (#3, #4, #9, and #18), pVL at first 100 ml peripheral blood extraction day was below or close to 5,000 copies of HIV RNA/ml, which may result from having a slow pVL rebound kinetics. VL rebound was particularly low in patient #9 perhaps reflecting partial post-ART control.

We chose to quantify viral production and immunogen concentration in our system as HIV RNA copies/ml, because it was previously reported that HIV-1 p24 antigen after heating was not properly identified by many commercial ELISAs (29). Nevertheless, an immunogen inactivation step is required by regulatory authorities to increase the safety of therapeutic vaccines employing immunogens generated from infectious autologous virus. No residual infectivity was evidenced after heat inactivation of the supernatants used to immunogen production in our HIV isolation procedure (Figure 5). Cycles of freezing–thawing (once pre- and twice during heat inactivation (Figure 1) contributed to further reducing potential HIV residual infectivity (30) without significantly affecting HIV RNA levels. This may be explained by a reduced size of the nucleic acid fragment amplified by qPCR and/or by a protection effect conferred by the virion capsid.

We were able to isolate HIV every time assays were performed (21 samples provided by 18 patients), demonstrating the consistency and robustness of this isolation approach. Two patients (#8 and #11) yielded autologous isolates not reaching 10^6^ HIV RNA copies/ml at the 7th coculture day and kept below that value 1 week later (Figure 3A). Therefore, in these two patients, new cocultures were performed to obtain better viral isolation yields (patients #8* and #11*). Despite three additional weeks without ART before new 100 ml blood collection (second isolation), low-level titers were again obtained from patient #11* whereas patient #8* was above threshold for both supernatants harvested. These data suggest that, for some patients, pVL may not be a reliable biomarker for *in vitro* HIV production. Good viral levels were obtained at coculture day 7 for patient #9, but viral load dropped at day 14 below 10^6^ HIV RNA copies/ml (Figure 3A). After supernatant concentration, 16 out of 18 patients (88.89% patients; no FP release for patients #8 and #11) initially recruited for this clinical trial yielded virus fulfilling criteria for immunogens as final products.

Overall, our method was of benefit for patients because STI interval was shortened in comparison with other techniques (Supplementary Table S1). The decrease of the viral load threshold to start HIV-1 isolation and the high production of virus in the aforementioned conditions reduced time of culture and allowed to restart antiretroviral treatment earlier.

It is noteworthy to notice that once the 7- and 14-day coculture supernatants contributed by the same donor at the same isolation were mixed, the resulting viral load is usually 1–2 logs higher than after concentration by ultracentrifugation (Figure 3C). This means that we have to improve our procedures in order to augment final product (FP) viral titer. Enhancing pellet formation and improving resuspension are crucial steps to that goal. The final immunogens exhibited a titer in the range of 10^10^ HIV RNA copies/ml (Figure 3; Supplementary Table S2), 47 times higher than our previous immunogen used in a prior anti-HIV therapeutic vaccine DCV2 clinical trial (13). This high immunogen amount used for pulsing of MDDC may boost host immune response at higher levels in comparison with previous studies (Supplementary Table S1).

The reason(s) why the coculture method provides virus yields higher than other previous HIV isolation approaches was not directly addressed in this study. The main difference with other isolation methods was coculture of monocytes and autologous CD4+ lymphocytes. Macrophages in cocultures might release microvesicles and/or secrete mediators that may facilitate virus production or relieve inhibition present in other culture approaches. The 1:1 proportion of macrophages and CD4^+^ lymphocytes could enhance immune synapse connections driving cellular activation and enhanced viral replication. The high virus yields we obtained may also be related to the recovery of the virus in both myeloid and lymphoid cells, although there appears to be limited number of virus in peripheral monocytes. Recent data on persistent virus reservoirs highlight the relevance of qualitative aspects of HIV proviruses, in particular the proportion of full-length replication-competent proviral genomes and the sites of integration (31). These aspects have not been studied in monocytes, and it remains an open possibility that the viral reservoir in macrophages could be enriched in replication-competent proviruses integrated in active transcriptional regions of the genome. Resistance of infected monocyte-derived macrophages infected *in vitro* to viral cytopathic effects could also contributed to increased virus yields.

Correlation analysis suggested that HIV production increased with augmenting MDM amount at viral culture onset (Figure 4A). Moreover, CD14^+^ cells and MDM number are positively related to pVL on the day of viral culture onset, suggesting that the patients with higher viral load could have an expansion of CD14^+^ monocytes in the blood (32, 33). Regarding the results on the negative correlation between age and HIV-1 production (Figure 4B), as well as age versus MDM (Figure 4C), we could speculate that increased age could reduce the number or differentiation of MDM by altering different CD subsets. In fact, increasing age is positively correlated with the CD14^low^CD16^+^ monocyte subset (34), which are less efficient at differentiating MDM compared with classical monocytes (35).

Viral production titers should be also dependent on the proportion of HIV-infected cells becoming transcriptionally active and producing HIV viruses. However, in our study, we did not find a correlation between viral production and total or integrated HIV-DNA. As previously mentioned, this could be due to a high proportion of defective viruses and low frequency of replication-competent integrated proviruses. Defective viruses can contribute to HIV RNA production (32) but do not generate viruses that are able to give rise to new infectious cycles. Interestingly, we have noticed that our HIV isolation procedure renders higher virus yields as measured by HIV-1 RNA but not commensurately increased, infectivity titers (lower TCDI50 values), suggesting the isolation of nonreplication-competent viruses and/or virus with lower affinity to HIV receptors. Another potential explanation to this lack of correlation could be due to integration of a high proportion of proviral copies in “silent” regions of the genome that are not readily accessible by the transcriptional machinery (36), generating what has been defined as “deep latency” (37, 38).

In summary, we have developed a novel approach for HIV isolation taking advantage of CD14^+^ and CD14^−^CD8^−^ cell fractions in PBMC collected from suppressed ART patients subjected to a brief structured treatment interruption. Using this methodological approach, we generate a final autologous virus produced in the patient-derived cells for DC loading, precluding potential issues related to alloreactivity. Viral stocks produced in short times under GMP conditions are suitable for production of an anti-HIV therapeutic DC-based vaccine. We speculate that the high titer of virus yield may be due to recovery of latent HIV in both myeloid and lymphoid cell fractions in the peripheral blood. Accordingly, using an *in vitro* cocktail of latency reversal agents (5) could further improve latent HIV depletion.

## Supplementary Material

Supp Fig 2 **Supplementary Figure 2** | Analysis of the association between VL HIV RNA (copies/mL) and amount of HIV p24 Antigen (ng/mL). HIV RNA and p24 antigen obtained from mixing supernatants collected after 7 and 14 days in the same coculture.

Supp Fig 1 **Supplementary Figure 1** | Detailed flow cytometry analysis of cultured cell subsets used for HIV-1 isolation. **(A)**. CD14^+^ monocytes differentiate to CD71^+^ monocyte-derived macrophages (MDM). **(B)** CD14^−^CD8^−^ cells were 24h-activated and harvested five days later, being CD25 determined. FSC, forward scatter; SSC, side scatter; PBMC, Peripheral Blood Mononuclear Cell; act, activated.

Supp Table 1 **Supplementary Table 1** | Summary of alternative methods for immunogen production for anti-HIV vaccines. The method reported for primary quantitation of viral yield (vRNA copies/mL, viral particles/mL, or HIV p24 ng/mL) is indicated in bold while to enable comparisions the corresponding values for the other parameters are also estimated, based on 2 vRNA copies/virion and 10^7^ virions/ng p24.

Supp Table 3 **Supplementary Table 3** | Additional viral p24 antigen data on ART patients subjected to HIV-1 isolation. Viral p24 antigen data on patient #9 was not determined. Patient.1 indicates when the isolation procedure was once more assayed. IQR (interquartile) percentiles as well as 25% and 75% percentiles are specified at the lower three positions in each column. ART, Antiretroviral Therapy; NA, Not Applicable. *, second 100mL peripheral blood extraction.

Supp Table 2 **Supplementary Table 2** | Additional data on ART patients subjected to HIV-1 isolation. Patient.1 indicates when the isolation procedure was once more assayed. IQR (interquartile) percentiles as well as 25% and 75% percentiles are specified at the lower three positions in each column. ART, Antiretroviral Therapy; VL, Viral Load; NA, Not Applicable. *, second 100mL peripheral blood extraction.

## Figures and Tables

**TABLE 1 |:** Clinical data on ART-treated patients subjected to HIV-1 isolation.

Patients	Age (years)	Years of infection	Years on ART	Peak of pVL (HIV RNA copies/ml)	CD4 nadir (cell/mm^3^)	HIV DNA at STI start (copies/10^6^ CD4+ T cell)		STI (days)	Peripheral blood extraction (100 ml)		
						Total	Integrate		CD4^+^ (cell/mm^3^)	CD8^+^ (cell/mm^3^)	pVL (HIV RNA copies/ml)
**# 1**	48	14.5	6.1	63,900	360	1,586.32	1,028.35	29	468	775	56,910
**# 2**	39	7.7	3.8	45,610	304	214.1	101.56	31	706	996	253,000
**# 3**	59	8.4	6.9	33,683	300	260.38	95.76	24	757	480	4,981
**# 4**	51	11.4	3.4	423,100	366	1,066.43	334.61	30	1,177	592	5,830
**# 5**	58	19.6	19.1	34,108	536	362.78	89.88	30	821	1,034	337,500
**# 6**	33	7.4	2.4	6,701	713	153.46	27.95	43	674	741	11,400
**# 7**	43	15.4	4.3	59,581	629	84.93	16.19	38	830	622	1,170,000
**# 8**	53	6.3	3	20,580	648	66.61	54.32	52	670	1,140	663,000
**# 8** *	NA	NA	NA	NA	NA	NA	NA	87	687	1,894	15,500
**# 9**	51	17.2	16.7	185,000	391	124.91	86	64	520	600	686
**# 10**	45	17.3	8.2	15,700	341	297.07	349.9	50	864	2,048	27,800
**# 11**	54	1.4	1	246,500	572	89.91	17.05	28	573	813	174,000
**# 11** *	NA	NA	NA	NA	NA	NA	NA	58	658	1,071	15,900
**# 12**	59	24	19	2,598	400	385.57	142.36	38	414	629	111,000
**# 13**	38	14.3	4	76,270	380	385.57	142.36	29	733	593	117,000
**# 14**	43	3.2	3	26,600	416	441.19	59.35	31	788	882	406,000
**# 14** *	NA	NA	NA	NA	NA	NA	NA	78	702	836	12,300
**# 15**	42	7.5	5.2	43,560	526	467.14	78.32	68	619	1,083	8,070,000
**# 16**	42	3.6	1.7	47,040	586	392.23	90.62	29	1,263	655	240,000
**# 17**	40	9.6	7.7	43,660	405	567.91	38.81	49	748	961	45,700
**# 18**	47	6.2	1.2	15,600	380	477.75	162.3	38	443	923	4,470
**Median**	**46**	**9**	**4.15**	**43,610**	**402.5**	**374.2**	**90.25**	**38**	**702**	**836**	**56,910**
**25th percentile**	41.5	6.275	2.85	19,360	364.5	146.3	50.44	29.5	596	625.5	11,850
**75th percentile**	53.25	15.85	7.825	66,993	575.5	469.8	147.3	55	804.5	1,053	295,250

# Patient 1 indicates when the isolation procedure was again assayed.

Interquartile (IQR) percentiles as well as 25th and 75th percentiles are specified at the lower three positions in each column.

ART, antiretroviral therapy; pVL, plasma viral load; STI, structured treatment interruption; NA, not applicable.

* Second 100 ml peripheral blood extraction.

Bold values are median ones.

## Data Availability

The original contributions presented in the study are included in the article/**Supplementary Material**. Further inquiries can be directed to the corresponding author.

## HIGHLIGHTS

- New autologous HIV immunogen production method for their use in therapeutic vaccines.
- High viral titers (>10^10^) obtained and faster generation of vaccine stocks.
- Immunological system boost in dendritic cell loaded with HIV therapeutic vaccines.
