# Supplementary figures and images for: Highly Efficient Autologous HIV-1 Isolation by Coculturing Macrophage With Enriched CD4+ T Cells From HIV-1 Patients
Source: Front Virol. Author manuscript; Available in PMC 2022 Oct 7. (PMC9364968; doi:10.3389/fviro.2022.869431)

Supplementary Fig. 2

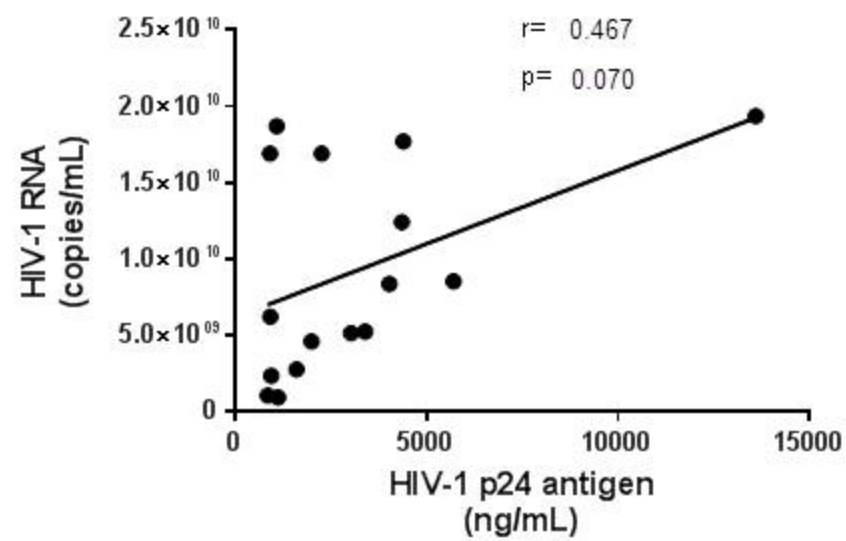

Supplement: Supp Fig 2 — Supplementary Figure 2 | Analysis of the association between VL HIV RNA (copies/mL) and amount of HIV p24 Antigen (ng/mL). HIV RNA and p24 antigen obtained from mixing supernatants collected after 7 and 14 days in the same coculture. [file NIHMS1796498-supplement-Supp_Fig_2.pdf]

Supplementary Fig 1

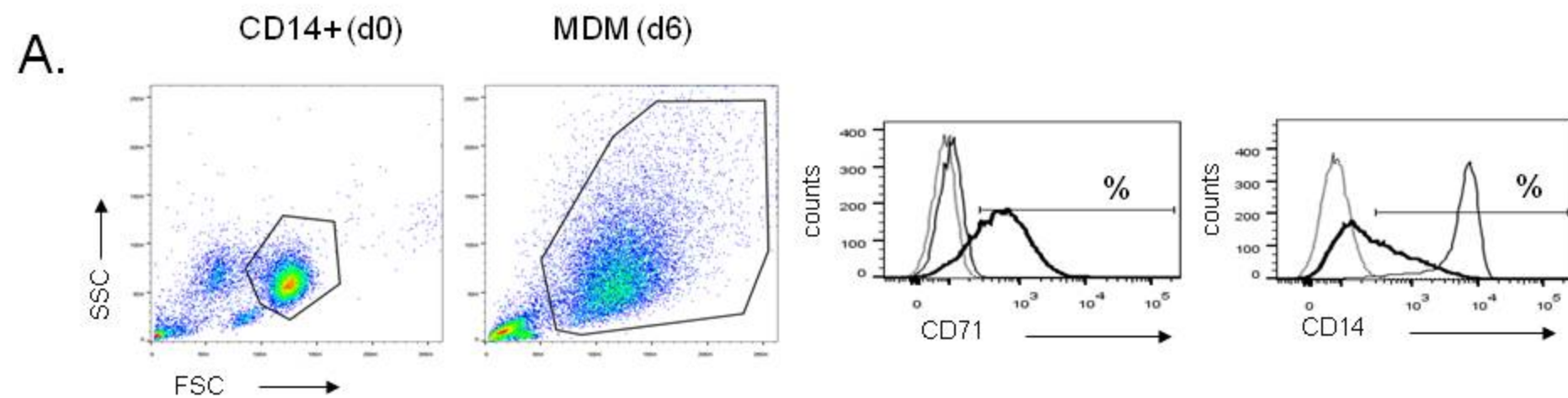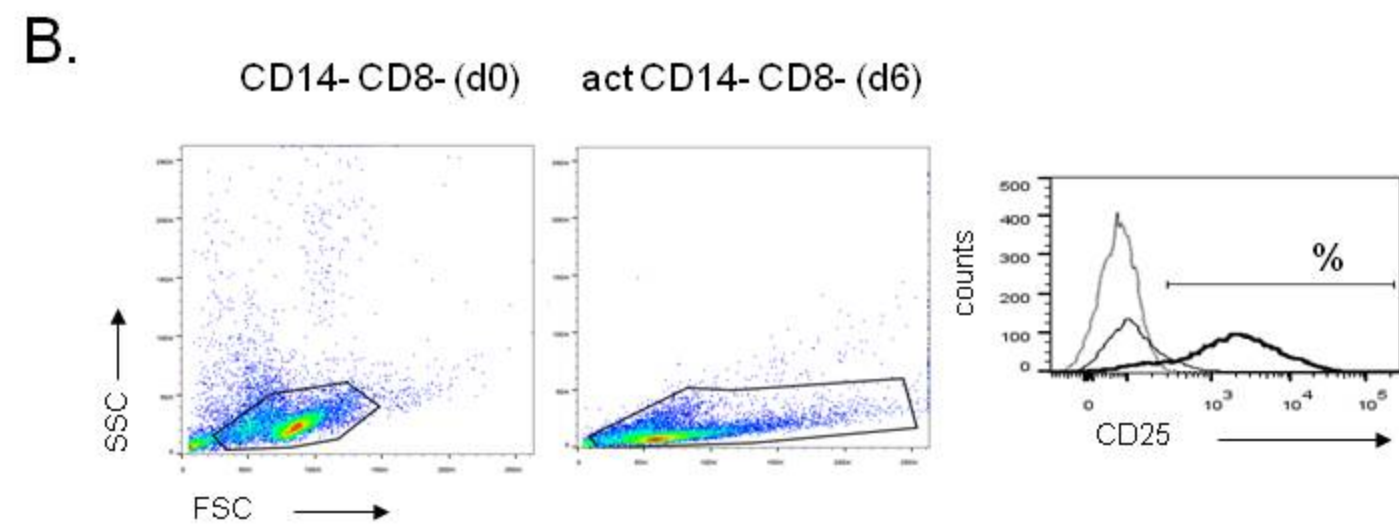

Supplement: Supp Fig 1 — Supplementary Figure 1 | Detailed flow cytometry analysis of cultured cell subsets used for HIV-1 isolation. (A). CD14+ monocytes differentiate to CD71+ monocyte-derived macrophages (MDM). (B) CD14−CD8− cells were 24h-activated and harvested five days later, being CD25 determined. FSC, forward scatter; SSC, side scatter; PBMC, Peripheral Blood Mononuclear Cell; act, activated. [file NIHMS1796498-supplement-Supp_Fig_1.pdf]
