# Supplementary material for: Highly Efficient Autologous HIV-1 Isolation by Coculturing Macrophage With Enriched CD4+ T Cells From HIV-1 Patients
Source: Front Virol. Author manuscript; Available in PMC 2022 Oct 7. (PMC9364968; doi:10.3389/fviro.2022.869431)
Supplement: Supp Table 1 — Supplementary Table 1 | Summary of alternative methods for immunogen production for anti-HIV vaccines. The method reported for primary quantitation of viral yield (vRNA copies/mL, viral particles/mL, or HIV p24 ng/mL) is indicated in bold while to enable comparisions the corresponding values for the other parameters are also estimated, based on 2 vRNA copies/virion and 107 virions/ng p24. [file NIHMS1796498-supplement-Supp_Table_1.pdf]

Supplementary Table 1: Summary of alternative methods for immunogen production for anti-HIV vaccines.

Ref.	pVL required to start HIV-1 isolation (RNA copies /mL)	Virus origin and spread	Time in culture (days)	HIV-1 Production (RNA copies /mL)
14	≥ 10000	Heterologous<br>Coculture of CD4+ T cells	30	$2x10^{9}$
15	> 5000	Plasmaphereses	1	$7,4x10^{8}$
16	≥ 50000	Leukaphereses<br>Superinfection of CD4+ enriched T cells from one healthy donor with autologous HIV-1 (Endogenous virus was isolated from peripheral blood-derived CD4+ T cells).	42-56	$6x10^{9}$
17; 29; 13	≥ 10000	150 mL peripheral blood<br>Heterologous coculture of CD4+ cells	28	$1.9x10^{9}$
18	Median 26225<br>(486-1860000)	Leukaphereses<br>Autologous culture of CD4+ cells	36-55	$4x10^{9}$
This manuscript	>5000	100 mL peripheral blood<br>Autologous coculture of CD14+ and CD4 + cells	20	$4,7x10^{10}$
