# Supplementary material for: Highly Efficient Autologous HIV-1 Isolation by Coculturing Macrophage With Enriched CD4+ T Cells From HIV-1 Patients
Source: Front Virol. Author manuscript; Available in PMC 2022 Oct 7. (PMC9364968; doi:10.3389/fviro.2022.869431)
Supplement: Supp Table 3 — Supplementary Table 3 | Additional viral p24 antigen data on ART patients subjected to HIV-1 isolation. Viral p24 antigen data on patient #9 was not determined. Patient.1 indicates when the isolation procedure was once more assayed. IQR (interquartile) percentiles as well as 25% and 75% percentiles are specified at the lower three positions in each column. ART, Antiretroviral Therapy; NA, Not Applicable. *, second 100mL peripheral blood extraction. [file NIHMS1796498-supplement-Supp_Table_3.pdf]

Supplementary Table 3

Patients	p24 day 7 (ng/mL)	p24 day 14 (ng/mL)	Vol. day 7 (mL)	Vol. day 14 (mL)	Vol. days 7+14 (mL)	p24 day 7 (ng)	p24 day 14 (ng)	p24 days 7+14 (ng total)
**# 1**	2410	1990	39.6	60	99.6	95400	119000	215000
**# 2**	3350	1000	44.9	68	113	150000	68300	219000
**#3**	488	632	18.5	28	46.5	9020	17700	26700
**# 4**	1320	676	15.8	24	39.8	20900	16200	37100
**# 5**	776	142	21.1	32	53.1	16400	4550	20900
**# 6**	317	774	58.1	88	146	18400	68100	86500
**# 7**	280	634	21.1	32	53.1	5910	20300	26200
**# 10**	1290	972	13.2	20	33.2	17000	19400	36400
**# 12**	490	459	37	56	93	18100	25700	43800
**# 13**	12500	1120	11.9	18	29.9	149000	20100	169000
**# 14**	2630	768	13.2	20	33.2	34700	15400	50100
**# 14***	2070	1960	26.4	40	66.4	54500	78400	133000
**# 15**	1200	406	68.6	104	173	82000	42200	124000
**# 16**	2790	2910	28.4	43	71.4	79200	125000	204000
**# 17**	1650	1380	63.4	96	159	104000	132000	237000
**# 18**	166	686	35.6	54	89.6	5910	37000	43000
**Median**	**1305**	**771**	**27.4**	**41.5**	**68.9**	**27800**	**31350**	**68300**
**25% Percentile**	488.5	632.5	16.48	25	41.48	16550	18125	36575
**75% Percentile**	2575	1315	43.58	66	109.7	92050	75875	195250
