# Supplementary material for: Highly Efficient Autologous HIV-1 Isolation by Coculturing Macrophage With Enriched CD4+ T Cells From HIV-1 Patients
Source: Front Virol. Author manuscript; Available in PMC 2022 Oct 7. (PMC9364968; doi:10.3389/fviro.2022.869431)
Supplement: Supp Table 2 — Supplementary Table 2 | Additional data on ART patients subjected to HIV-1 isolation. Patient.1 indicates when the isolation procedure was once more assayed. IQR (interquartile) percentiles as well as 25% and 75% percentiles are specified at the lower three positions in each column. ART, Antiretroviral Therapy; VL, Viral Load; NA, Not Applicable. *, second 100mL peripheral blood extraction. [file NIHMS1796498-supplement-Supp_Table_2.pdf]

Supplementary table2

Patients	VL day 7 (HIV-1 RNA copies/mL)	VL day 14 (HIV-1 RNA copies/mL)	Vol. day 7 (mL)	Vol. day 14 (mL)	Vol. Days 7 + 14 (mL)	Total VL day 7 (HIV-1 RNA copies)	Total VL day 14 (HIV-1 RNA copies)	Total VL days 7+ 14 (HIV-1 RNA copies)	FP (HIV-1 RNA copies)
**# 1**	$9.10x10^{9}$	$8.60x10^{9}$	39.6	60	99.6	$3.60x10^{11}$	$5.16x10^{11}$	$8.76x10^{11}$	$5.80x10^{10}$
**# 2**	$1.16x10^{10}$	$8.10x10^{8}$	44.9	68	113	$5.21x10^{11}$	$5.51x10^{10}$	$5.76x10^{11}$	$6.00x10^{10}$
**#3**	$6.80x10^{8}$	$2.40x10^{8}$	18.5	28	46.5	$1.26x10^{10}$	$6.72x10^{9}$	$1.93x10^{10}$	$1.00x10^{9}$
**# 4**	$3.50x10^{9}$	$1.10x10^{9}$	15.8	24	39.8	$5.53x10^{10}$	$2.64x10^{10}$	$8.17x10^{10}$	$1.20x10^{10}$
**# 5**	$5.80x10^{9}$	$4.24x10^{8}$	21.1	32	53.1	$1.22x10^{11}$	$1.36x10^{10}$	$1.36x10^{11}$	$2.97x10^{9}$
**# 6**	$8.00x10^{9}$	$1.07x10^{10}$	58.1	88	146	$4.65x10^{11}$	$9.42x10^{11}$	$1.41x10^{12}$	$4.50x10^{10}$
**# 7**	$5.10x10^{9}$	$1.18x10^{10}$	21.1	32	53.1	$1.08x10^{11}$	$3.78x10^{11}$	$4.85x10^{11}$	$1.30x10^{10}$
**# 8**	$2.72x10^{4}$	$3.80x10^{5}$	18.5	28	46.5	N.A	N.A	N.A	N.A.
**# 8***	$8.58x10^{6}$	$1.30x10^{7}$	17.2	26	43.2	$1.48x10^{8}$	$3.38x10^{8}$	$4.86x10^{8}$	N.A.
**# 9**	$6.50x10^{6}$	$6.10x10^{5}$	24	36	60	$1.56x10^{8}$	N.A	$1.56x10^{8}$	$1.50x10^{8}$
**# 10**	$5.10x10^{9}$	$1.18x10^{10}$	13.2	20	33.2	$6.73x10^{10}$	$2.36x10^{11}$	$3.03x10^{11}$	$1.75x10^{9}$
**# 11**	$2.58x10^{5}$	$3.60x10^{5}$	10.6	16	26.6	N.A	N.A	N.A.	N.A.
**# 11***	$1.79x10^{5}$	$9.20x10^{4}$	7.92	12	19.9	N.A	N.A	N.A.	N.A.
**# 12**	$4.10x10^{8}$	$1.94x10^{9}$	37	56	93	$1.52x10^{10}$	$1.09x10^{11}$	$1.24x10^{11}$	$5.95x10^{10}$
**# 13**	$5.97x10^{9}$	$1.34x10^{10}$	11.9	18	29.9	$7.10x10^{10}$	$2.41x10^{11}$	$3.12x10^{11}$	$1.38x10^{10}$
**# 14**	$4.20x10^{9}$	$1.05x10^{9}$	13.2	20	33.2	$5.54x10^{10}$	$2.10x10^{10}$	$7.64x10^{10}$	N.A.
**# 14***	$5.48x10^{9}$	$2.89x10^{9}$	26.4	40	66.4	$1.45x10^{11}$	$1.16x10^{11}$	$2.60x10^{11}$	$8.95x10^{10}$
**# 15**	$2.09x10^{9}$	$6.74x10^{8}$	68.6	104	173	$1.43x10^{11}$	$7.01x10^{10}$	$2.13x10^{11}$	$1.17x10^{11}$
**# 16**	$3.93x10^{9}$	$4.60x10^{9}$	28.4	43	71.4	$1.12x10^{11}$	$1.98x10^{11}$	$3.09x10^{11}$	$1.44x10^{11}$
**# 17**	$3.58x10^{9}$	$1.56x10^{9}$	63.4	96	159	$2.27x10^{11}$	$1.50x10^{11}$	$3.77x10^{11}$	$1.53x10^{11}$
**# 18**	$4.26x10^{8}$	$6.22x10^{8}$	35.6	54	89.6	$1.52x10^{10}$	$3.36x10^{10}$	$4.88x10^{10}$	$2.61x10^{10}$
**Median**	**$3.58x10^{9}$**	**$1.05x10^{9}$**	**21.1**	**32**	**53.1**	**$8.95x10^{10}$**	**$1.90x10^{11}$**	**$2.36x10^{11}$**	**$3.56x10^{10}$**
**25% Percentile**	**$2.09x10^{8}$**	**$1.27x10^{8}$**	**14.5**	**22**	**36.5**	**$1.52x10^{10}$**	**$2.37x10^{10}$**	**$6.95x10^{10}$**	**$5.23x10^{9}$**
**75% Percentile**	**$5.64x10^{9}$**	**$6.60x10^{9}$**	**38.3**	**58**	**96.3**	**$1.65x10^{11}$**	**$2.38x10^{11}$**	**$4.04x10^{11}$**	**$8.21x10^{10}$**
